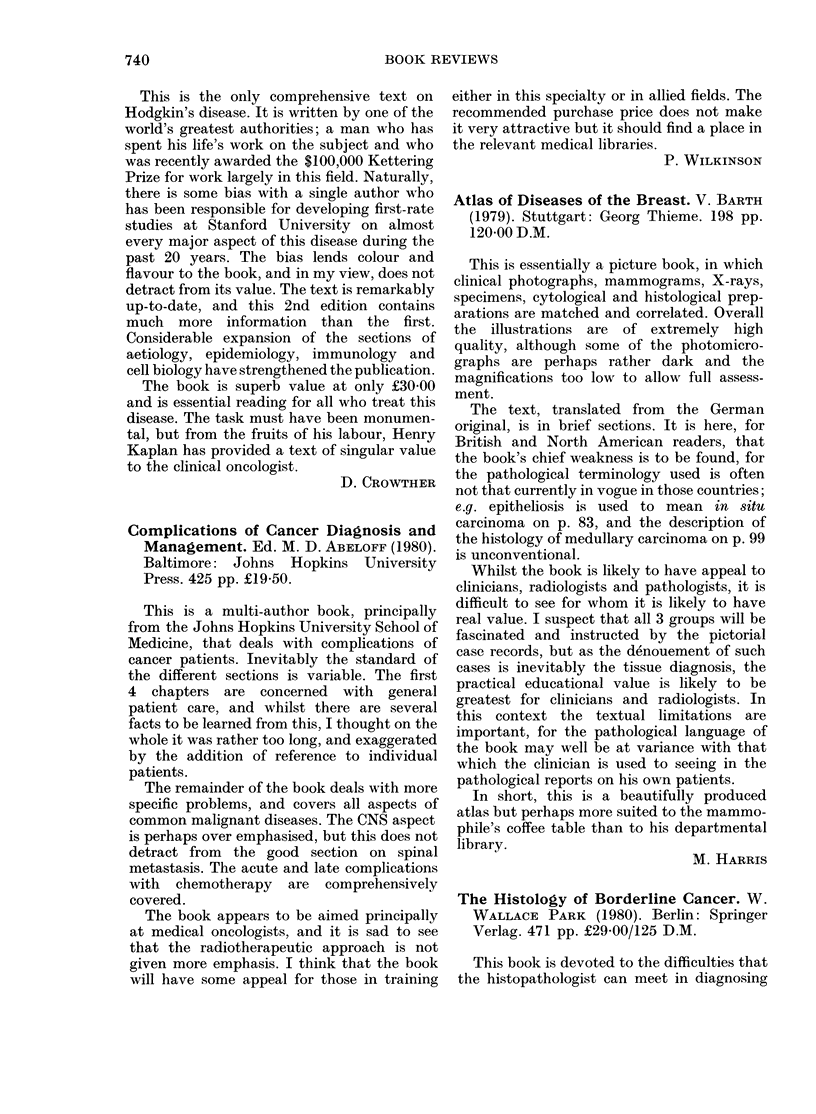# Atlas of Diseases of the Breast

**Published:** 1981-05

**Authors:** M. Harris


**Atlas of Diseases of the Breast.** V. BARTH (1979). Stuttgart: Georg Thieme. 198 pp. 120·00 D.M.

This is essentially a picture book, in which clinical photographs, mammograms, X-rays, specimens, cytological and histological preparations are matched and correlated. Overall the illustrations are of extremely high quality, although some of the photomicrographs are perhaps rather dark and the magnifications too low to allow full assessment.

The text, translated from the German original, is in brief sections. It is here, for British and North American readers, that the book's chief weakness is to be found, for the pathological terminology used is often not that currently in vogue in those countries; *e.g.* epitheliosis is used to mean *in situ* carcinoma on p. 83, and the description of the histology of medullary carcinoma on p. 99 is unconventional.

Whilst the book is likely to have appeal to clinicians, radiologists and pathologists, it is difficult to see for whom it is likely to have real value. I suspect that all 3 groups will be fascinated and instructed by the pictorial case records, but as the dénouement of such cases is inevitably the tissue diagnosis, the practical educational value is likely to be greatest for clinicians and radiologists. In this context the textual limitations are important, for the pathological language of the book may well be at variance with that which the clinician is used to seeing in the pathological reports on his own patients.

In short, this is a beautifully produced atlas but perhaps more suited to the mammophile's coffee table than to his departmental library.

M. HARRIS